# Artificial Intelligence (AI) Assisted Brief Psychological Intervention for Adolescents with Complex Emotional Needs

**DOI:** 10.1192/bjo.2026.11184

**Published:** 2026-06-30

**Authors:** Manusmriti Acharya, Liza Farhana Ferdousi, Brioney Gee, Joanna Baines, Franco Orsucci

**Affiliations:** 1 Norfolk and Suffolk Foundation Trust, Bury St Edmunds, United Kingdom; 2 University of East Anglia, Norwich, United Kingdom; 3 Norfolk and Suffolk Foundation Trust, Norwich, United Kingdom; 4 University of Amsterdam, Amsterdam, Netherlands

## Abstract

**Aims:**

The aim of this study is to design and create a virtual coach powered by artificial intelligence (AI) to support young people aged 14–17 who are receiving a brief talking therapy for complex emotional needs (CEN).

Creating an AI-driven virtual coach to use in-between the sessions offers a potential solution to enhance engagement, accessibility and outcome without replacing the human therapist.

**Methods:**

We planned three work packages over a 10-month period starting from July 2025.

Work package 1 focused on understanding the needs and preferences of the AI coach’s intended users and those who will be influential in its adoption and sustainment, including young people aged 14–17 with CEN, their parents/carers and youth mental health clinicians. Semi-structured qualitative interviews and focus groups were carried out, transcribed verbatim and analysed thematically using a framework approach.

Work package 2 focused on design and creation of an initial prototype of the AI coach based on the findings of work package one. A code design workshop was planned where the participants advisory group worked with the research team to agree on how the AI coach should be structured and operate to support the brief psychological intervention.

The objective of work package 3 is to refine the prototype AI coach in preparation for future research. Participants who took part in work package 1 will be invited to interact with the prototype AI coach for the purpose of providing feedback. A bespoke feedback form will be designed to encourage participants to critique all relevant aspects of the prototype. This feedback will be used to produce a refined version which participants will again be invited to review. The refined prototype will also undergo technical and clinical review including for equitable performance across demographic groups.

**Results:**

Participants viewed the virtual coach as a feasible and acceptable adjunct to traditional care. Based on the themes from work package 1, an initial prototype of the AI coach based is created. We aim to further refine the prototype AI coach in preparation for future research.

**Conclusion:**

We believe an AI-powered virtual coach can improve accessibility, engagement and outcome for adolescents receiving brief interventions. Based on the findings, the research team will work further to agree plans for future research mostly to evaluate clinical outcomes and address ethical considerations in AI-driven mental health support.

